# Microscale Investigation of Arsenic Distribution and Species in Cement Product from Cement Kiln Coprocessing Wastes

**DOI:** 10.1155/2013/518676

**Published:** 2013-10-07

**Authors:** Yufei Yang, Jingchuan Xue, Qifei Huang

**Affiliations:** ^1^State Key Laboratory of Environmental Criteria and Risk Assessment, Chinese Research Academy of Environmental Science, Beijing 100012, China; ^2^Wadsworth Center, New York State Department of Health, and Department of Environmental Health Sciences, School of Public Health, State University of New York at Albany, Empire State Plaza, P.O. Box 509, Albany, NY 12210-0509, USA

## Abstract

To improve the understanding of the immobilization mechanism and the leaching risk of Arsenic (As) in the cement product from coprocessing wastes using cement kiln, distribution and species of As in cement product were determined by microscale investigation methods, including electron probe microanalysis (EPMA) and X-ray absorption spectroscopy. In this study, sodium arsenate crystals (Na_3_AsO_4_12H_2_O) were mixed with cement production raw materials and calcined to produce cement clinker. Then, clinker was mixed water to prepare cement paste. EPMA results showed that As was generally distributed throughout the cement paste. As content in calcium silicate hydrates gel (C-S-H) was in low level, but higher than that in other cement mineral phases. This means that most of As is expected to form some compounds that disperse on the surfaces of cement mineral phases. Linear combination fitting (LCF) of the X-ray absorption near edge structure spectra revealed that As in the cement paste was predominantly As(V) and mainly existed as Mg_3_(AsO_4_)_2_, Ca_3_(AsO_4_)_2_, and Na_2_HAsO_4_.

## 1. Introduction 

In many developing countries, the use of cement kilns to coprocess wastes containing heavy metals is thriving and plays an exceedingly important role in solid waste, especially hazardous waste disposal [1–3]. Coprocessed wastes include electroplating sludge, contaminated soil, chromium slag, sludge, hazardous combustible liquid wastes, and garbage [2]. During coprocessing, almost all nonvolatile and semivolatile heavy metals are transferred into cement clinker which causes the heavy metal concentration in the cement to increase significantly. These heavy metals in cement will be released into the environment gradually and cause a new environmental risk, and this risk is being taken seriously [4].

The release behaviors of heavy metals from cements and cementitious materials, as well as the release mechanisms and influence factors, have been studied to evaluate the environmental risk [5–10]. Actually, the distribution and the specific species of heavy metals in cement products are crucial for evaluating the environmental pollution risk because these affect the release behaviors of heavy metals greatly. 

Most pieces of research previously conducted were focused on the distribution and the specific species of heavy metals in cement-based solidified/stabilized wastes [11–13]. Jing et al. have analyzed the arsenic species and components in cement-based solidified/stabilized wastes [14, 15]. Some studies revealed that the immobilization of arsenic with cement and lime is generally attributed to the formation of insoluble calcium arsenic compounds, such as CaHAsO_3_ for arsenite containing wastes [16] and Ca_3_(AsO_4_)_2_ for S/S treated As(V)-bearing samples [17, 18].

Although the distribution and the specific species of heavy metals in cement-based solidified/stabilized wastes were conducted, the study on the coprocessing cement product was limited. As the physical and chemical properties will be changed during calcination processes, the distribution and the specific species of heavy metals in the coprocessing cement product will be different from that in cement-based solidified/stabilized wastes [19]. Therefore, it is essential to study the distribution and the specific species of heavy metals in the cement product from cement kiln coprocessing of wastes.

In this paper, a microscale investigation of arsenic in cement clinker and paste made with cement from simulated coprocessing wastes using cement kiln was conducted. Electron probe microanalysis (EPMA) was used to determine the As distribution and its associations with cement mineral phases, while X-ray absorption near edge structure (XANES) spectroscopy was used to obtain detailed information on the As valence state and major compounds in the cement clinker and paste. This information will be useful for evaluating the environmental pollution risk.

## 2. Materials and Methods

### 2.1. Raw Material Characterization

Raw cement materials, including limestone, clay, and iron powder, were obtained from a local cement plant, and their chemical compositions are listed in Table 1. The As contents in the raw materials, the amount of chemical reagents added in the experiment, and their corresponding ratios for cement production, which were calculated based on the arsenic content, are listed in Table 2.

### 2.2. Sample Preparation

Na_3_AsO_4_·12H_2_O was evenly mixed with the cement raw materials in accordance with the ratios listed in Table 2. A mass fraction of 1% distilled water was added to the mixture. After stirring regularly, disk samples (ø80 mm × 15 mm thick) were prepared. The disks were oven dried at 105°C and then calcined at 1450°C for 1 h. Finally, the disks were rapidly cooled to room temperature in air. A portion of the clinkers were taken out and stored for XANES analysis.

A mass fraction of 5% gypsum was added to the remaining clinkers. Then the mixture was ground to fine cement consisting of particles with specific surface area of 310 m^2^/kg. Later, cement paste test pieces (20 × 20 × 10 mm) were made at a water to cement ratio of 0.3. They were first cured at 20°C and 96% relative humidity for 24 hour and then demolded. Finally, they were cured at (20 ± 2)°C and 95% relative humidity for 28 d prior to testing. Some of the cured cement test pieces were crushed in a jaw crusher until 95% of the sample was <125 *μ*m in size for XANES analysis.

### 2.3. Electron Probe Microanalysis

The remaining cement paste test pieces were placed in an oven at 60°C for 1-2 h. Once dry, the sample was soaked in epoxide-resin glue, and the temperature was increased to 50–60°C so that the epoxide-resin glue filled the spaces within the sample. Then the sample was placed in a vessel under a low vacuum to remove any air and cured in an oven at 60°C for 4 h. A diamond saw was used to cut smaller samples from the original sample, and then these cut samples were ground and polished for analysis by EPMA.

The microstructures were identified with the help of energy-dispersive X-ray spectroscopy (EDS, Oxford ISIS300), which is used to determine elemental composition. A JEOL JXA8800R EPMA analyzer was also used. The voltage employed was 15 keV, and the electron beam current was 2 × 10^−8^ A. BSE images and X-ray images (elemental distribution images) were acquired. National EPMA oxides and silicate standard samples were used. 

The overall features of the cement mineral phase were first observed with the low power lens (scale of 200 *μ*m), and then typical mineral phases were transferred to the middle lens (scale of 50–100 *μ*m) for EPMA surface, line, and point analyses. Some mineral phases with fine particles were transferred to the high power lens (scale of 20 *μ*m) for these analyses.

### 2.4. XANES Measurements

Arsenic K-edge XANES spectra were collected at the Beijing Synchrotron Radiation Facility (BSRF). The typical energy of the storage ring was 2.5 GeV with the current decreasing from 250 to 160 mA during runs. Mg_3_(AsO_4_)_2_, Ca_3_(AsO_4_)_2_, Na_2_HAsO_4_, Na_3_AsO_4_, and NaAsO_2_ were recorded as standards in transmission mode (TM). Two samples, clinker and cement paste, were measured in fluorescence mode (FM). All standards were ground to fine grains and pressed to form wafers (ø1 mm). Data analyses of the experimental XANES spectra were performed with FEFF8.0 [20].

## 3. Results and Discussion

### 3.1. Distribution and Association with Specific Mineral Phases

**Figure 1:**
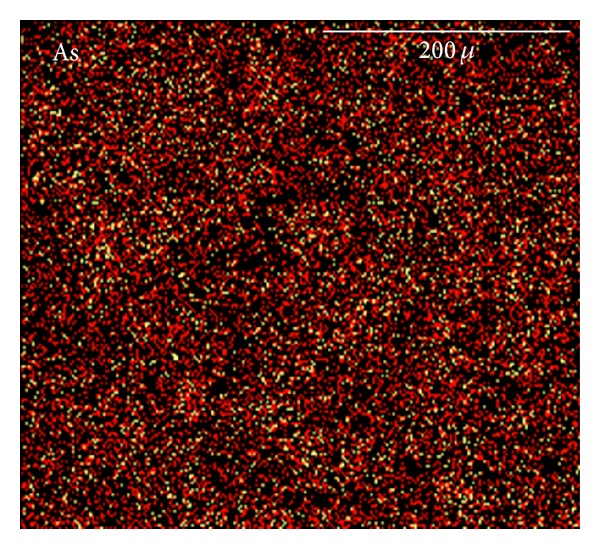
X-ray image of As in cement paste.

The elemental distribution image of As obtained with EPMA using the low-power lens is shown in Figure 1. Bright areas corresponding to high concentrations of As were very small and could not be distinguished easily. What also can be found is that the As is generally distributed throughout the cement paste.

**Figure 2:**
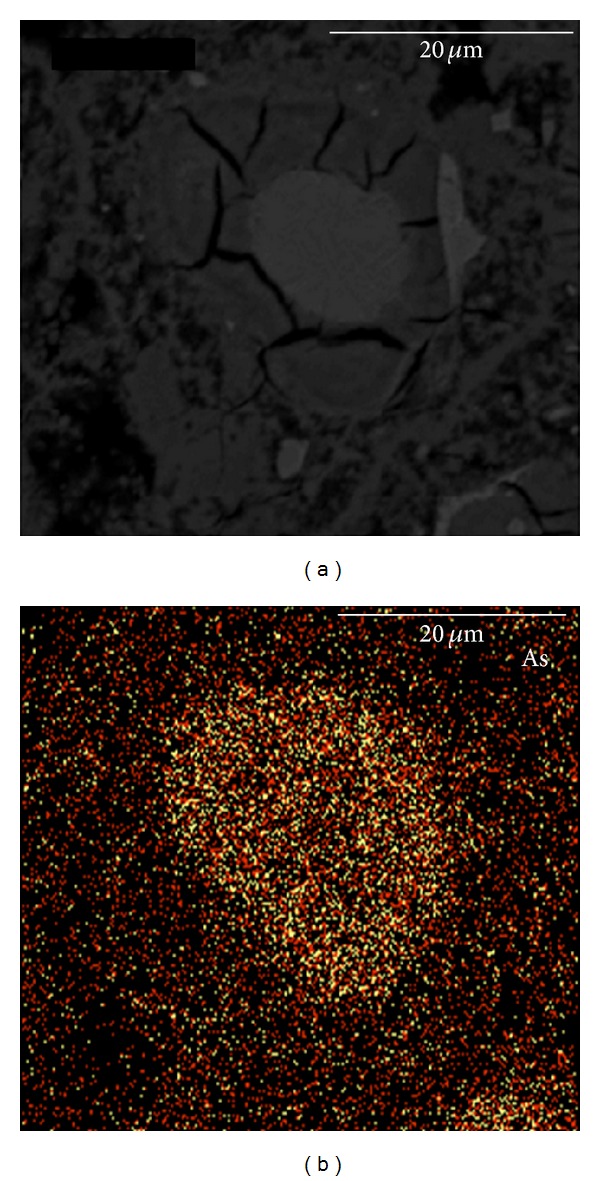
Typical feature of C-S-H gels in cement paste and the corresponding X-ray image of As.

**Figure 3:**
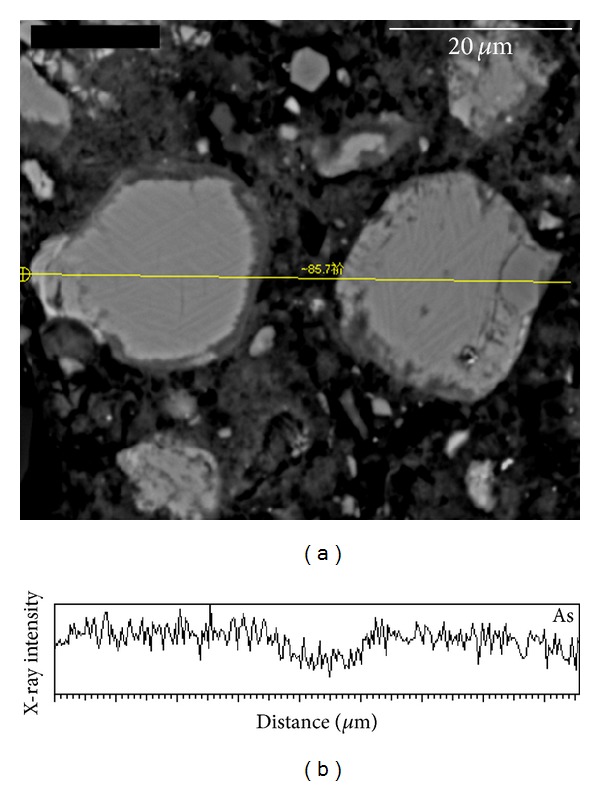
EPMA line analysis of two blocks of C-S-H gels in cement paste and relevant results of As.

BSE-imaging allows minerals of different compositions to be identified.  Figure 2(a) shows a typical calcium silicate hydrates (C-S-H) gel feature. The elemental distribution map (Figure 2(b)) showed that the arsenic content was slightly associated with the C-S-H gels. Line analysis (Figure 3) of other typical C-S-H gel features confirmed this correlation.

**Figure 4:**
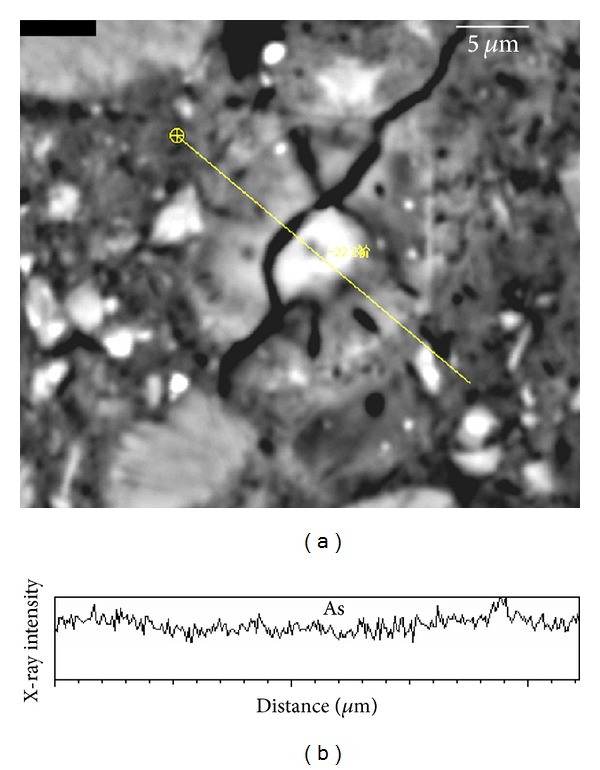
EPMA line analysis of CH crystal in cement paste and corresponding results of As.

Figure 4(a) shows the BSE image of a typical portlandite (CH crystal), and Figure 4(b) is the X-ray intensity curve along the line in Figure 4(a). This indicates that the arsenic content in the CH crystal is very low. The EDS analyses (Table 3) of random spots on the CH crystal confirmed this.

The EDS analyses (Table 4) of random spots on the intermediate phases, including calcium aluminates and calcium ferrites, indicated that their arsenic content was low.

A tentative explanation for the weak associations of arsenic with cement mineral phases is that most arsenic forms some arsenates dispersed on the surfaces of hydrates. These arsenates were mostly not incorporated into the hydrates in the calcination and hydration processes, and they became independent mineral phases adhered on the surfaces of hydrates. The association of arsenic with C-S-H phase maybe due to the high binding ability to arsenic compounds of C-S-H gels.

### 3.2. Specific Species of Arsenic

**Figure 5:**
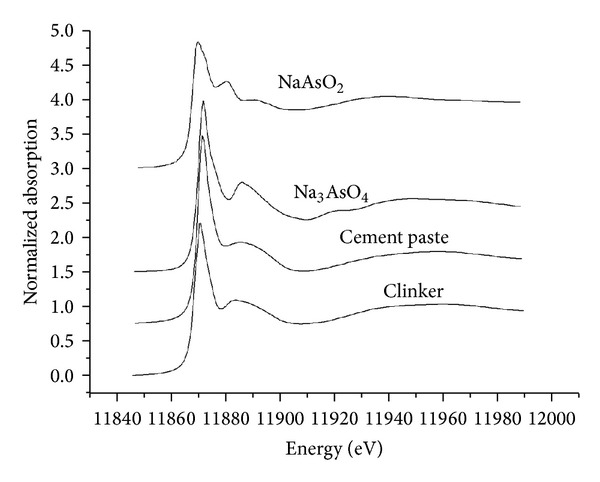
As K-edge XANES spectra for the samples and two standard compounds.

Arsenic K-edge XANES spectra of the samples and two standards are shown in Figure 5. The initial sharp peak in the XANES spectra arises from a transition of the excited photoelectron from the 1*s* level to vacant 4*p* levels. Huffman et al. [21] found that the energy of the *s*–*p* peak increased along with the valence state of the arsenic. Manning et al. [22, 23] indicated that the excitation energy for As(III) was well separated from that of As(V) by about 4 eV. In the present study, the peak energies for the cement paste and clinker were the same as that of sodium arsenate, in which the arsenic has a valence state of +5, and higher than that of sodium arsenite, in which the arsenic has a valence state of +3. This indicates that the arsenic in the cement paste and clinker has a valence state of +5. 

The spectra of cement paste and clinker showed similar general trends. However, there were some interesting differences. For example, the *s*–*p* peak for the clinker was obviously more intense and narrower than that for the cement paste (Figure 5). Additionally, some secondary structures were visible from 11880–11900 eV for the clinker, but not evident for the cement paste. This suggests that the arsenic compounds in the clinker are crystalline.

**Figure 6:**
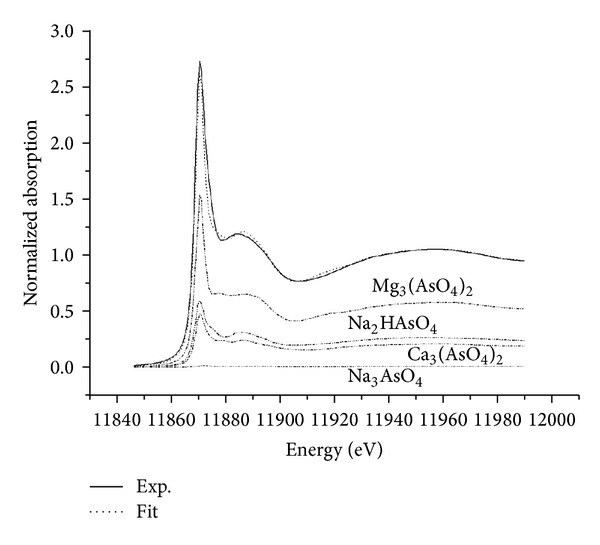
Near edge spectra of the cement paste compared with a sum of 55% Mg_3_(AsO_4_)_2_, 25% Na_2_HAsO_4_, 19.4% Ca_3_(AsO_4_), and 0.5% Na_3_AsO_4_. The small differences between the two spectra are likely due to the disordered environment.

Using the spectra of standards, LCF has been successfully applied to identify and quantify the main components of cement-immobilized materials [14]. In this work, LCF was performed on the XANES data, and the fitting range was 144 eV around the absorption edge (about 11846–11990 eV). The fitting results are shown in Figure 6.

The main component in the cement paste was Mg_3_(AsO_4_)_2_ with a mass fraction of 55%. The other major components were Na_2_HAsO_4_ and Ca_3_(AsO_4_)_2_ with mass fractions of 25% and 19.4%, respectively. By contrast, the content of Na_3_AsO_4_, which was the initial chemical added during production of the cement sample, was very low in the final product. Because Mg_3_(AsO_4_)_2_ and Ca_3_(AsO_4_)_2_ are stable compounds, most of the arsenic present in coprocessed cement can be immobilized. 

## 4. Conclusions 

Most of the arsenic in cement clinker and paste was present as more stable compounds, such as Mg_3_(AsO_4_)_2_ and Ca_3_(AsO_4_)_2_, which is formed in the cement production process. 

Arsenic compounds in the clinker were similar to those in the cement paste, but they were crystalline instead of amorphous. This indicates that these compounds are mainly generated in the calcination process, and the hydration reaction contributes little to their formation. These stable compounds result in the immobilization of arsenic in coprocessed products. 

Arsenic compounds mainly disperse on the surfaces of the hydrates, only the C-S-H phase contained arsenic among the cement mineral phases, which may be due to the high binding ability to arsenic compounds of C-S-H gels.

## Figures and Tables

**Table 1:** Chemical composition of raw materials (%).

Items	SiO_2_	Fe_2_O_3_	Al_2_O_3_	CaO	MgO	LOSS
Limestone	0.18	0.04	0.04	55.64	0.05	43.40
Clay	62.14	9.10	16.17	1.63	0	7.30
Ion powder	34.98	50.56	4.95	1.34	0.84	4.52

**Table 2:** Design and materials in experiment.

Items	As
Ni content in the raw material^1^/(mg·kg^−1^)	81.6
Chemical reagents added	Na_3_AsO_4_·12H_2_O
Adding ratio^1^/%	0.3
Adding amount in the raw material^1^/g·kg^−1^	17

^1^Calculated by *w.*

**Table 3:** EPMA point analysis data of CH crystal in cement paste.

Number	Element content (%)				
	Al	Si	Ca	Fe	As
1	0.09	0.85	60.47	0.65	0
2	0.05	0.79	56.78	0.66	0.03
3	0.15	1.07	57.69	0.45	0.28
4	0	2.56	36.32	0.62	0.52
5	0.20	2.71	37.78	0.68	0.42
6	0.13	1.16	54.44	0.79	0
7	0.27	1.49	57.05	0.66	0
8	0.27	1.25	56.79	0.59	0
9	0.43	3.06	54.23	0.70	0

**Table 4:** EPMA point analysis data of middle phases containing Fe, Al in cement paste.

Number	Element content (%)				
	Al	Si	Ca	Fe	As
1	17.21	6.12	50.80	15.16	0
2	19.70	4.39	50.19	16.69	0
3	20.27	4.06	48.71	17.08	0
4	20.77	4.15	47.93	16.82	0
5	19.28	4.03	50.23	17.96	0
6	21.70	4.18	48.05	17.42	0
7	20.54	4.21	50.54	16.60	0
8	19.35	4.06	51.66	15.25	0
9	21.39	4.36	47.57	16.97	0
10	20.91	4.30	47.39	15.14	0
